# Impact of Gastrointestinal Side Effects on Patients’ Reported Quality of Life Trajectories after Radiotherapy for Prostate Cancer: Data from the Prospective, Observational Pros-IT CNR Study

**DOI:** 10.3390/cancers13061479

**Published:** 2021-03-23

**Authors:** Marianna Noale, Alessio Bruni, Luca Triggiani, Michela Buglione, Filippo Bertoni, Luca Frassinelli, Rodolfo Montironi, Renzo Corvò, Vittorina Zagonel, Angelo Porreca, Pierfrancesco Bassi, Mauro Gacci, Giario Natale Conti, Stefania Maggi, Stefano Magrini, The Pros-IT CNR Study Group

**Affiliations:** 1 National Research Council, Neuroscience Institute, Aging Branch, 35128 Padua, Italy; marianna.noale@in.cnr.it (M.N.); stefania.maggi@in.cnr.it (S.M.); 2 Radiotherapy Unit, University Hospital of Modena, 41124 Modena, Italy; brunialessio@virgilio.it (A.B.); lucafrassinelli@yahoo.it (L.F.); 3 Radiation Oncology Department, University and Spedali Civili Hospital, 25123 Brescia, Italy; michela.buglione@unibs.it (M.B.); stefano.magrini@unibs.it (S.M.); 4 Prostate Group of the Italian Association for Radiation Oncology (AIRO), 20124 Milan, Italy; filippo.bertoni@gmail.com; 5 Section of Pathological Anatomy, Polytechnic University of the Marche Region, 60126 Ancona, Italy; r.montironi@staff.univpm.it; 6 Department of Radiation Oncology, Ospedale Policlinico San Martino and University of Genoa, 16132 Genoa, Italy; renzo.corvo@unige.it; 7 Medical Oncology Unit, Veneto Institute of Oncology IOV-IRCCS, 35128 Padua, Italy; vittorina.zagonel@iov.veneto.it; 8 Urological Oncology, Veneto Institute of Oncology IOV-IRCCS, 35128 Padua, Italy; angeloporreca@gmail.com; 9 Department of Urology, Catholic University of Rome, Policlinico Gemelli, 00168 Rome, Italy; bassipf@gmail.com; 10 Department of Urologic Robotic Surgery and Renal Transplantation, Careggi Hospital, University of Florence, 50134 Florence, Italy; maurogacci@yahoo.it; 11 Urology Unit, Azienda Socio-Sanitaria Territoriale Lariana, Sant’Anna Hospital, 22042 Como, Italy; giario.conti@gmail.com

**Keywords:** prostate cancer, health-related quality of life, growth mixture model, radiation therapy

## Abstract

**Simple Summary:**

The analyses presented, based on data from the PROState cancer monitoring in ITaly from the National Research Council (Pros-IT CNR) study, evaluated patients’ reported quality of life outcomes related to bowel function and bother over a 2-year period from the diagnosis. Growth mixture models were considered for patients who underwent radiotherapy, including treatments that were associated or not associated with androgen deprivation therapy. Our data revealed that the proportion of patients that were radiotherapy-treated with persisting significant bowel worsening at the 24 months interval is very limited. The absence of comorbidities and the use of intensity or volumetric radiotherapy techniques with image guidance were found to be related with a better tolerance to radiotherapy in terms of bowel side effects. These findings could be relevant for treatment selection and accurate patient information.

**Abstract:**

Radiotherapy (RT) represents an important therapeutic option for the treatment of localized prostate cancer. The aim of the current study is to examine trajectories in patients’ reported quality of life (QoL) aspects related to bowel function and bother, considering data from the PROState cancer monitoring in ITaly from the National Research Council (Pros-IT CNR) study, analyzed with growth mixture models. Data for patients who underwent RT, either associated or not associated with androgen deprivation therapy, were considered. QoL outcomes were assessed over a 2-year period from the diagnosis, using the Italian version of the University of California Los Angeles-Prostate Cancer Index (Italian-UCLA-PCI). Three trajectories were identified for the bowel function; having three or more comorbidities and the use of 3D-CRT technique for RT were associated with the worst trajectory (OR = 3.80, 95% CI 2.04–7.08; OR = 2.17, 95% CI 1.22–3.87, respectively). Two trajectories were identified for the bowel bother scores; diabetes and the non-Image guided RT method were associated with being in the worst bowel bother trajectory group (OR = 1.69, 95% CI 1.06–2.67; OR = 2.57, 95% CI 1.70–3.86, respectively). The findings from this study suggest that the absence of comorbidities and the use of intensity modulated RT techniques with image guidance are related with a better tolerance to RT in terms of bowel side effects.

## 1. Introduction

Radiotherapy (RT) represents an important therapeutic option for the treatment of localized prostate cancer (PCa), as was also confirmed in recently published on-going prospective studies investigating patterns of practice and related toxicities [1,2]. The choice between RT and surgery may depend on different variables (such as comorbidities, risk of toxicities, patient preference and/or life expectancy) and cancer features so that the definitive adoption of a therapeutic option need to be accompanied by complete information about advantages and side effects of each choice, particularly about urinary, bowel, and sexual function [3].

Despite the increasing value of newer imaging tools and the advances of the RT techniques and technologies that allow us to precisely deliver higher target doses and simultaneously spare surrounding normal tissues, pelvic RT may still be challenging due to the exposition of healthy organs (organs at risk, OARs) to radiation [4]. Indeed, image guided Intensity modulated RT (IG-IMRT) is actually widely diffused (using different tools such as Cone Beam CT, fiducials markers, and MRI integrated tools) representing a recommended tool when high doses (conventional or hypofractionated) have to be delivered. However, several patients may experience different acute/subacute side effects such as diarrhea, abdominal pain, and urgency that could represent an important risk factor for also developing consequential late bowel symptoms. Late effects may develop months to years later, being—although rarely—permanent and progressive in severity, thus also affecting the quality of life (QoL) of the patients [5].

Research data on the deterioration of QoL in PCa patients treated with RT have been based on group means, which very often do not provide insights into subgroup trajectories. Growth mixture models (GMMs) are a method for identifying multiple unobserved sub-populations, describing longitudinal changes within each sub-population. In other words, changes over time of some parameters can be used for post-hoc identification of sub-populations and to describe the differences noted between them [6].

Previous analyses based on the same patient population of the present one showed that acute bowel toxicity was frequent among RT treated patients, although most of them recovered at the 24 months follow up interval [7]. We therefore hypothesized that different trajectories of patients’ reported QoL gastrointestinal side effects exist, and that specific characteristics of patients at diagnosis might be associated with each trajectory. The aim of the current analyses was to examine trajectories in bowel function and bowel bother (BF, BB) in males diagnosed with PCa undergoing RT enrolled in and being monitored within the Pros-IT CNR project over a 2-year period from the time of diagnosis.

## 2. Materials and Methods

### 2.1. Participants

Pros-IT CNR is a longitudinal, observational study that has been monitoring QoL in a sample of Italian patients diagnosed with biopsy-verified PCa [8]. The 97 Urology, Radiation, and Oncological Italian facilities participating in the project enrolled 1705 treatment-naïve patients between September 2014 and September 2015 [9]. Data regarding the patients’ demographic and anamnestic characteristics, diagnosis, cancer clinical staging, risk factors, comorbidities, and health-related QoL scores were collected during the baseline assessment held at the time of diagnosis. Data on cancer treatments and QoL scores were evaluated and reported at each of the scheduled follow-up evaluations held 6, 12, 24, 36, 48, and 60 months after the time of diagnosis [10]. Only the data of the patients who underwent RT, whether associated or not associated with androgen deprivation therapy (ADT), were considered in the current report.

The approval of the Ethics Committee of the coordinating center (Sant’Anna Hospital, Como, Italy; register number 45/2014) and of all the hospitals or health care facilities involved in the project was obtained. All the participants signed informed consent forms. The study was carried out in accordance with the principles of the Declaration of Helsinki.

### 2.2. Outcome Variables

QoL outcomes were assessed using validated questionnaires that were self-administered at the time patients were diagnosed and during the follow-up assessments; RT patients’ QoL over a 2-year period from the time of diagnosis was considered in the current analyses. Bowel function and bother (BF and BB, respectively) were evaluated using the Italian version of the University of California Los Angeles-Prostate Cancer Index (Italian-UCLA-PCI available at https://eprovide.mapi-trust.org/instruments/ucla-prostate-cancer-index (accessed on 25 January 2021) in the original English version and in other 13 languages, including the Italian validated version) [11]. The scores ranged from 0 to 100, with higher scores indicating better outcomes.

The study’s endpoints were related to changes in BF and BB scores from the time of diagnosis to the 24-month follow-up evaluation in PCa patients submitted to RT, whether associated or not associated with ADT.

### 2.3. Predictor Variables

Socio-demographic variables at diagnosis, clinical variables including comorbidities (evaluated using the Cumulative Illness Rating Scale [12]), the Gleason score, clinical T score, prostate specific antigen (PSA) at diagnosis, and the PCa treatments that were carried out over the 24-month follow-up period examined here were considered predictors.

### 2.4. Statistical Analysis

The participants who completed one or more follow-up assessments were included in the analysis; no imputation of missing values was performed.

The summary statistics were expressed as means ± standard deviation (SD) or median and Quartile 1 (Q1), Quartile 3 (Q3)) for quantitative variables and frequency percentages for categorical variables.

Group-based modeling was implemented using SAS Proc Traj to identify subgroups of participants sharing similar QoL trajectories [13]. This technique identifies the distinct patterns for the dependent variables (QoL measures) over time. The best model was selected by considering:

- The model fit statistics in terms of the Bayesian Information Criterion (BIC); the magnitude of the BIC difference was utilized to choose between more complex and simpler models (2ΔBIC > 10 [13]);
- the significance of polynomial terms, starting with a cubic specification for the trajectory shape and dropping non-significant polynomial terms [14];
- the value of group membership probability of at least 5%;
- the value of average posterior probability within each group greater than 0.7.

Chi-square, Kruskal-Wallis tests, and univariate logistic regression models were used to evaluate the unadjusted differences among the trajectory groups identified. As a sensitivity analysis, group-based modeling considering only patients completing the 24-month follow-up was evaluated.

Multivariable logistic regression models were used to evaluate variables associated with the trajectory groups. Independent variables were selected using a stepwise selection procedure (a *p*-value of 0.10 was set to entry and a *p*-value to stay 0.20). Age at diagnosis, education, marital status, family history of prostate cancer, comorbidities, diabetes, body mass index (BMI), prostate-specific antigen (PSA) at diagnosis, Gleason score, clinical T-Stage, and characteristics of RT (method: Image-Guided Radiation Therapy-IGRT vs. non-IGRT), technique (three-dimensional conformal radiotherapy (3D-CRT) vs. Intensity-Modulated Radiation Therapy (IMRT), Volumetric Modulated Arc Therapy (VMAT) and Stereotactic body radiotherapy (SBRT)), the volume treated (prostate alone vs. prostate + seminal vesicles vs. prostate + seminal vesicles + pelvic nodes), association with ADT treatments (only RT, ADT before RT, ADT after RT), and RT dose were considered to be possible independent variables.

Statistical significance was assessed using a 2-sided *p* < 0.05. Analyses were performed using SAS software version 9.4.

## 3. Results

**Figure 1:**
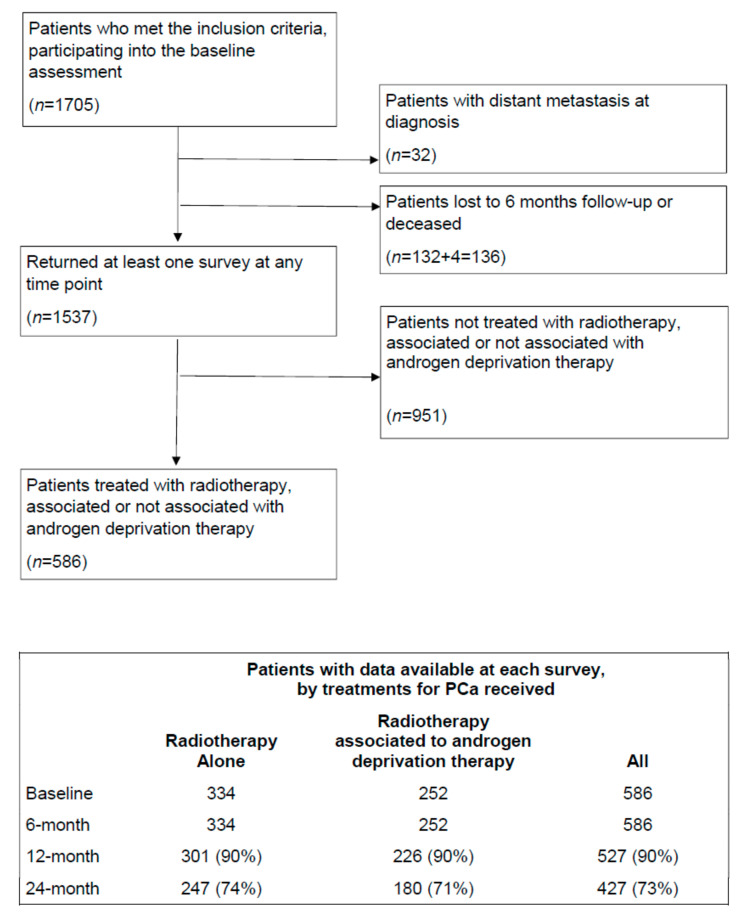
Flow diagram of the analytic cohort considered in the present analysis.

One thousand seven hundred and five patients were enrolled in the Pros-IT CNR study; their characteristics at diagnosis have been described in detail elsewhere [9]. Data on PCa treatments were available for 1537 patients (Figure 1); only the data regarding the patients who underwent RT (*n* = 334) and RT plus ADT (*n* = 252) were analyzed here (total patients = 586). Five hundred twenty-seven participants (90%) took part in the 12-month follow-up assessment and 427 (73%) in the 24-month one. Patients lost to follow-ups did not significantly differ from those who participated to the follow-up assessments in relation to age and Italian-UCLA-PCI scores at diagnosis (*p* > 0.05).

The “overall” column of Table 1 describes patients’ features at diagnosis and characteristics of RT; their mean age was 72.7 years; 19% of them presented 3 or more moderate, severe, or extremely severe diseases, and almost 20% had diabetes mellitus. The median PSA level at diagnosis was 7.9 ng/mL (Q1 = 5.4; Q3 = 11.5), and 65% of the participants had a clinical stage ≥ T2. The Gleason score at diagnosis was 6 for 35% of the study participants, 3 + 4 for 26%, 4 + 3 for 16% and ≥8 for 23%. Four hundred and twenty-three (75%) patients underwent IGRT while 143 (25%) non-IGRT methods. A 3D-CRT technique was used in 186 patients (33%), IMRT in 229 (40%), VMAT in 143 (26%), and SBRT in 9 (1%). The prostate alone, the prostate + seminal vesicles, and the prostate + seminal vesicles + pelvic nodes were treated respectively in 143 (26%), 324 (59%), and 86 patients (15%). Exclusive RT was used in 333 patients (59%); ADT was administered before or during RT in 107 (18%), while it was also administered after the RT end in 135 (23%).

The responses the participants gave to questions on the Italian-UCLA-PCI regarding BF and/or BB at the time of diagnosis were available for 572 (2.4% missing data); the mean value for BF was 91.8 ± 15.2; it was 91.8 ± 20.3 for BB.

**Figure 2:**
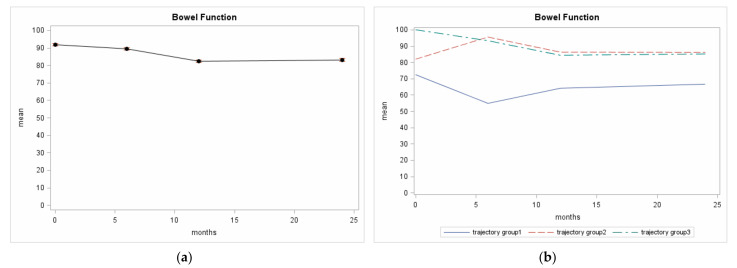
Italian- University of California Los Angeles-Prostate Cancer Index (Italian-UCLA-PCI) BF scores from Pros-IT participants across 4 evaluations; (**a**) Overall mean and standard error; (**b**) BF trajectory patterns identified.

Overall, the mean BF scores decreased during the first 12 months after diagnosis and then tended to stabilize until the 24-month follow-up (Figure 2a). Three trajectories were identified for the BF scores over the 24-month period after diagnosis (Figure 2b):

- Trajectory 3 referred to the largest group of patients (67% of the population considered in the present analysis) who had high BF scores at diagnosis (the mean baseline score was 100 ± 0) that fell gradually throughout the 24-month follow-up (mean score at the 6-month follow-up: 93.2 ± 13.1, at the 12-month follow-up: 84.4 ± 14.3, at the 24-month follow-up: 85.3 ± 12.8). The posterior group membership probability was 0.9;
- Trajectory 2 referred to the second largest group of patients (23%) whose BF scores were lower at diagnosis (the mean baseline score was 78 ± 14), but higher at the 6-month follow-up assessment (mean score: 95.6 ± 7.6); they fell again (mean score at the 12-month follow-up: 85.9 ± 10.2) and were lower by the time the 24-month follow-up assessment was reached (mean score: 86.5 ± 10.2). The posterior group membership probability was 0.9;
- Trajectory 1 referred to the smallest group of patients (10%) who started with lower scores (the mean baseline score was 71 ± 23); it initially fell (mean score at the 6-month follow-up: 52.1 ± 23.2) and then rose remaining at a plateau until the 24-month follow-up assessment was reached (mean score at the 12-month follow-up: 62.9 ± 23.7, at the 24-month follow-up: 63.2 ± 23.5). The posterior group membership probability was 0.9.

With the exception of the number of comorbidities, the three groups of patients showed non-significant unadjusted differences in characteristics at diagnosis (Table 1). The difference between trajectory 2 and 3 at presentation (Figure 2b), may be due to the different *spectra* of comorbidities before PCa treatments; data after treatment, however, are almost overlapping. In comparison to patients following trajectories 2 and 3, those on trajectory 1 have worse BF at presentation, and even worse BF at the six-month follow-up, probably due to acute RT toxicity in patients submitted to less sophisticated treatments, then only partially recovering.

The prevalence of patients with three or more moderate/severe/very severe comorbidities was more than double in the Trajectory 1- with respect to the Trajectory 3-patient group (Odds Ratio (OR) for trajectory 1 vs. 3 from univariate model 3.33 (95% Confidence Interval (CI) 1.84–6.04). RT was performed with IMRT or VMAT technique in 53% of the Trajectory 1-patients, in 62% of the Trajectory 2-patients and in 70% of the Trajectory 3-patients (OR for trajectory 1 vs. 3: 0.48, 95% CI 0.28–0.84, OR for trajectory 2 vs. 3 0.70: 95% CI 0.46–1.08). IGRT was used in 63% of the Trajectory 1-patients, in 72% of the Trajectory 2-patients, and in 78% of the Trajectory 3-patients (OR for non IGRT for trajectory 1 vs. 3: 2.00, 95% CI 1.12–3.57, OR for trajectory 2 vs. 3: 1.35, 95% CI 0.86–2.11). There were non-significant differences in the three groups with regard to the association of ADT to RT: 68% of the Trajectory 1-patients had RT alone, 17% had ADT before RT, and 15% had ADT after RT; 55%, 18%, and 28% of the Trajectory 2-patients respectively underwent those treatments, as did respectively 58%, 19%, and 23% of the Trajectory 3-patients.

Results from group-based modeling considering only patients completing the 24-month follow-up (sensitivity analysis) were overlapping with those from the overall population (Table S1).

The patient characteristics in the multivariable logistic model that were significantly associated with the trajectories according to the stepwise selection procedure were number of comorbidities and RT technique (Table S2). In particular, having three or more moderate/severe/very severe comorbidities and undergoing RT performed with 3D-CRT technique were associated with the Trajectory 1 vs. Trajectory 3 groups (OR = 3.80, 95% CI 2.04–7.08, *p* < 0.0001; OR = 2.17, 95% CI 1.22–3.87, *p* = 0.0088, respectively). Borderline significant associations were found for these variables in the Trajectory-2 vs. Trajectory-3 patients (OR = 1.67, 95% CI 0.99–2.83, *p* = 0.0560; OR = 1.47, 95% CI 0.94–2.28, *p* = 0.0881, respectively). A borderline significant association was also found in relation to the use of ADT after RT (OR = 0.47, 95% CI 0.21–1.07, *p* = 0.0731 for Trajectory 1 vs. 3).

**Figure 3:**
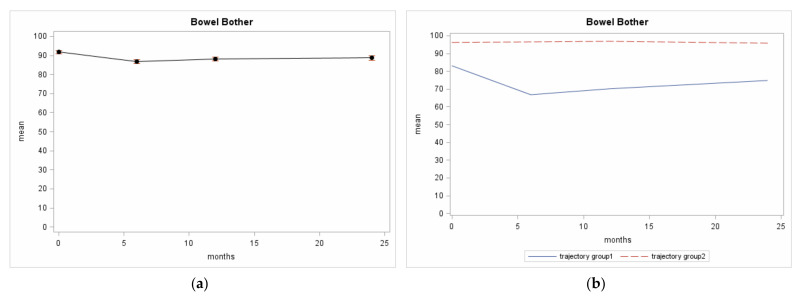
Italian-UCLA-PCI BB scores from Pros-IT participants across 4 evaluations; (**a**) Overall mean and standard error; (**b**) BB trajectory patterns identified.

The BB scores showed a plateau effect in the entire population throughout the 24-month period following diagnosis (Figure 3a). Two trajectories were identified for the BB score patterns over the 24 month follow-up period studied (Figure 3b):

- Trajectory 2 referred to the largest proportion of the patients (71% of those considered) who showed constantly high BB scores throughout the 24-month follow-up period studied (96 ± 13 was the mean baseline BB score, 98 ± 9 was the score at the 6-and 98 ± 8 at the 12-month follow-ups, and 96 ± 14 at the 24-month follow-up). The posterior group membership probability was 0.9;
- Trajectory 1 referred to a smaller group of patients (29%) who showed lower BB scores at diagnosis (the mean baseline score was 81 ± 29) and even lower scores at the 6-month follow-up (61 ± 30); then the curve stabilized or rose slightly until the 24-month follow-up assessment was reached (64 ± 30 at the 12-month follow-up; 73 ± 31 at the 24-month follow-up). The posterior group membership probability was 0.8.

With the exception of diabetes, there were no significant unadjusted differences in the variables at diagnosis between the Trajectory 1- and Trajectory 2-patients (Table 2). The prevalence of diabetes was 25% in the Trajectory 1-patients and 18% in the Trajectory-2 patients (OR from univariate model 1.59, 95% CI 1.04–2.45). There were no significant unadjusted differences with regard to the RT technique (37% of the Trajectory-1 patients underwent 3D-CRT vs. 32% of the Trajectory 2-patients; OR = 1.03, 95% CI 0.88–1.21) and to the association of RT and ADT (OR = 1.08, 95% CI 0.67–1.72 and OR = 0.91, 95% CI 0.98–1.42 for ADT before RT and ADT after RT, respectively, vs. only RT). IGRT was used in 62% of the Trajectory 1-patients and in 80% of the Trajectory 2-ones (OR for non-IGRT = 2.51, 95% CI 1.69–3.74, from a univariate model). The patient characteristics in the multivariable logistic model significantly associated with the trajectory groups according to the stepwise selection procedure were having diabetes and the RT methods (Table S4). Diabetes and the non-IGRT method were both associated with being in the Trajectory 1-group vs. the Trajectory 2-group (OR = 1.69, 95% CI 1.06–2.67, *p* = 0.0269; OR = 2.57, 95% CI 1.70–3.86, *p* < 0.0001, respectively).

Sensitivity analysis considering only patients completing the 24-month follow-up confirmed the results from the overall population (Table S3).

## 4. Discussion

In our analysis, three different subgroup of patients (trajectories) were identified in terms of bowel functions tolerance and consequently patients QoL perception. At the time of diagnosis, all different subgroups showed similar features in terms of most of patient, tumor, and therapies characteristics. However, further investigations revealed that the presence of three or more comorbidities before being submitted to RT seems to be correlated with worse BF scores determining faster worsening of QoL (trajectory 3 vs. 1, *p* < 0.0002).

These findings are substantially in line with the study from Geinitz et al. [15] in which the presence of comorbidities at time of diagnosis seems to be a predictor of impaired QoL after PCa RT concomitant to ADT. This is a relevant observation because, as was already reported in a previous analysis from Pros-IT CNR investigation, patients referred to RT facilities are often older and frailer than surgical ones, with a consequent impaired baseline QoL. This previous analysis from the Pros-IT CNR study showed that more advanced age (OR = 6.1, *p* < 0.0001) was one of the independent predictors of receiving RT (instead of surgery) as first radical therapeutic approach. On the other hand, a better Physical Composite Score (PCS) as for the Short-Form Health Survey (SF-12) was linked to an inferior probability of receiving RT [16]. This insight may be of interest for the multidisciplinary team discussion involving also urologists and medical oncologists. Often these patients are referred to radiation oncologists due to their general status, but it is important to underline with other specialists and patients themselves that performance status or comorbidities may result in a significative worsening of common acute and late side effects known to be RT related. However, also in the older age groups, the fraction of RT-treated patients with persisting significant BB of BF worsening at the 24 months interval is very limited. Moreover, younger, more fit patients may be offered high dose RT with a minimal risk of persisting BF and BB.

These findings are in line with the available literature. Hamstra et al. [17] reported how comorbidities may negatively influence the clinical tolerance to dose escalated RT, particularly in terms of late rectal toxicity. A higher Charlson Comorbidity Index appeared to be strongly related with rectal toxicity, especially when a medical history of either myocardial infarction or congestive heart failure was present before RT. In addition, the common use of oral anticoagulants seems to increase side effects independently of the patient’s age and number of comorbidities [17]. Similarly, patients with diabetes mellitus reported a lower BB scores both at diagnosis (the mean baseline score was 81) and even lower scores at the 6-month follow-up, demonstrating once more how the presence of endocrine systemic disease may affect the overall tolerance to RT treatment. Recently this link was also confirmed by an interesting review showing how the interaction between pro-inflammatory cytokines such as IL1, IL-6, IL-18, and TNF-11, through oxidative stress mechanisms and MPO, MDA, IL-1, and TNF-alfa production, induced by ionizing radiation, may explain the excess of toxicities reported in several diabetic patients [18]. Recently, the impact of diabetes on radiation-induced gastrointestinal toxicity was also investigated in a clinical retrospective study on adjuvant RT for gynecological malignancies driven by Ozkan EE et al. [19]. Through the analysis of more than 100 patients affected by endometrial or cervical carcinoma treated mostly with 3D CRT, authors found that the incidence of proctitis was significantly lower in patients who were not diabetic (*p* < 0.04) and confirmed that diabetes increased the risk of radiation toxicity, reset the onset of symptoms to an earlier time, and worsened its resolution [20].

A second relevant observation is that more sophisticated RT techniques such as IMRT or VMAT using the IGRT technique seem to be more likely linked with better trajectories (3 in terms of BF, 2 for BB). In particular, the 3D-CRT technique was associated with the trajectory 1 with a risk twice as high (OR = 2.17, 95% CI 1.23–3.87, *p* = 0.0088) of BF worsening up to a 24-month follow up. Similarly, delivering RT without image guidance appears to double the risk (OR = 2.57, 95% CI 1.70–3.86, *p* < 0.0001) of developing a significative impairment in terms of BB. The role of new RT technologies is still debated. A second relevant observation is that more sophisticated RT techniques such as IMRT or VMAT using the IGRT technique seem to be linked with better trajectories (3 for BF, 2 for BB). In particular, the 3D-CRT technique was associated with the trajectory 1 with a double higher risk (OR = 2.17, 95% CI 1.23–3.87, *p* = 0.0088) of BF worsening up to a 24-month follow-up. Similarly, delivering RT without image guidance appears to double the risk (OR = 2.57, 95% CI 1.70–3.86, *p* < 0.0001) of developing impairment in terms of BB. The role of new RT technologies is still debated, but the majority of the papers published on this issue seems to provide evidence of an advantage for the use of IGRT, in particular when the increased accuracy in dose delivery has been exploited to reduce CTV-PTV margins, independently from the use of fiducial markers [21,22,23,24,25].

The current study has some strengths and limitations. A strength is certainly the prospective population-based design, including contemporary patients enrolled only for around a year. This feature indeed yielded a cohort of patients that is more representative of a national scenario than institutional reports. Furthermore, to our knowledge, this is the first study that investigated the correlation between population features and RT related toxicity through sophisticated statistical models such as the growth mixture models and trajectories. On the other side, the study has some limitations: first, the participating centers were involved on a voluntary basis, thus we cannot exclude a selection bias. Second, the observational design of the study has made our analysis susceptible to confounders, even if the use of sophisticated statistical models should have reduced major uncertainties. Third, 90% and 73% of patients that were initially enrolled participated in the 12- and the 24-month follow-ups, and this may have influenced the trajectories analysis; however, we verified that quality of life scores at diagnosis of participants lost to follow-ups were not significantly different from those of patients who participated until the 24-month follow-up. Fourth, further studies on different and larger cohorts, with longer follow-ups need to be considered to validate the results of the present analyses.

## 5. Conclusions

Our analysis on patients submitted to high dose RT with radical intent showed that no comorbidities and the use of IGRT might be linked to better BB and BF patient reported outcomes. These findings may be relevant in multidisciplinary meeting discussions where the risks and benefits of each treatment should be clearly discussed between the specialists and then referred to patients to make the optimal choice in the era of a tailored approach.

## Figures and Tables

**Table 1:** Characteristics of patients according to trajectories identified for bowel function (BF).

Characteristics of Patients	Overall (*n* = 586)	Trajectory 1 (*n* = 60)	Trajectory 2 (*n* = 134)	Trajectory 3 (*n* = 392)	*p*-Value
Age at diagnosis, years, mean ± SD	72.7 ± 5.5	71.9 ± 5.6	72.5 ± 6.3	72.9 ± 5.2	0.4244
Education > lower secondary school, *n* (%)	241 (42.0)	24 (40.0)	56 (42.1)	161 (42.3)	0.9468
BMI ≥ 30 kg/m^2^, *n* (%)	106 (18.8)	14 (23.3)	28 (21.4)	64 (17.1)	0.4072
Current smoker, *n* (%)	72 (12.5)	7 (11.9)	18 (13.7)	47 (12.2)	0.8922
Diabetes mellitus, *n* (%)	116 (19.9)	15 (25.0)	28 (21.1)	73 (18.7)	0.4887
3+ moderate/severe comorbidities *, *n* (%)	109 (18.6)	22 (36.7)	29 (21.8)	58 (14.8)	0.0002
Family history of prostate cancer, *n* (%)	76 (13.3)	7 (11.7)	20 (15.0)	49 (12.7)	0.7363
T staging at diagnosis, *n* (%)					0.2689
T1	194 (34.8)	15 (26.8)	44 (35.8)	135 (35.6)	
T2	266 (47.7)	32 (57.2)	63 (51.2)	171 (45.1)	
T3 or T4	98 (17.6)	9 (16.1)	16 (13.0)	73 (19.3)	
Gleason score at diagnosis, *n* (%)					0.8773
≤6	203 (35.0)	23 (39.7)	45 (33.6)	135 (34.8)	
3 + 4	151 (26.0)	14 (24.1)	34 (25.4)	103 (26.6)	
4 + 3	93 (16.0)	11 (19.0)	20 (14.9)	62 (16.0)	
≥8	133 (22.9)	10 (17.2)	35 (16.1)	88 (22.7)	
PSA at diagnosis, ng/mL, median (Q1, Q3)	7.9 (5.4, 11.5)	8.4 (5.5, 10.9)	8 (5.3, 12.4)	7.8 (5.4, 11.3)	0.2366
D’Amico risk class, *n* (%)					0.9117
Low	80 (13.9)	6 (10.3)	20 (15.3)	54 (14.0)	
Intermediate	235 (40.8)	26 (44.8)	52 (39.7)	157 (40.6)	
High	261 (45.3)	26 (44.8)	59 (45.0)	176 (45.4)	
RT method, *n* (%)					0.0445
IGRT	423 (74.7)	38 (63.3)	95 (72.0)	290 (77.5)	
Non-IGRT	143 (25.3)	22 (36.7)	37 (28.0)	84 (22.5)	
RT technique, *n* (%)					0.0006
3D-CRT	186 (32.8)	28 (46.7)	47 (35.6)	111 (29.6)	
IMRT	229 (40.4)	18 (30.0)	48 (36.4)	163 (43.5)	
VMAT	143 (25.2)	14 (23.3)	30 (22.7)	99 (26.4)	
SBRT	9 (1.5)	0 (0.0)	7 (5.3)	2 (0.5)	
Volume treated with RT, *n* (%)					0.4779
Prostate alone	143 (25.9)	14 (23.3)	30 (23.4)	99 (27.1)	
Prostate plus seminal vesicles	324 (58.6)	35 (58.3)	83 (64.8)	206 (56.4)	
Prostate, seminal vesicles and pelvic nodes	86 (15.5)	11 (18.3)	15 (11.7)	60 (16.4)	
Dose Gy, *n* (%)					0.1798
<70	105 (21.3)	7 (12.1)	27 (22.9)	71 (22.4)	
70–75	210 (42.6)	26 (44.8)	43 (36.4)	141 (44.5)	
>75	178 (36.1)	25 (43.1)	48 (40.7)	105 (33.1)	
Association RT and ADT, *n* (%)					0.3554
Only RT	333 (57.9)	41 (68.3)	73 (54.5)	227 (58.4)	
ADT before or during RT	107 (18.6)	10 (16.7)	24 (17.9)	73 (18.8)	
ADT after RT	135 (23.5)	9 (15.0)	37 (27.6)	89 (22.9)	

SD: Standard Deviation; BMI: Body Mass Index; Q1: Quartile 1; Q3: Quartile 3; RT: Radiotherapy; IGRT: Image-Guided Radiation Therapy; 3D-CRT: three-dimensional conformal radiotherapy; IMRT: Intensity-Modulated Radiation Therapy; VMAT: Volumetric Modulated Arc Therapy; SBRT: Stereotactic body radiotherapy; * Based on Cumulative Illness Rating Scale (CIRS).

**Table 2:** Characteristics of patients according to the trajectories identified for BB.

Characteristics of Patients	Overall (*n* = 586)	Trajectory 1 (*n* = 170)	Trajectory 2 (*n* = 416)	*p*-Value
Age at diagnosis, years, mean ± SD	72.7 ± 5.5	72.3 ± 6.0	72.5 ± 6.3	0.4543
Education > lower secondary school, *n* (%)	241 (42.0)	79 (47.0)	162 (39.9)	0.1157
BMI ≥ 30 kg/m^2^, *n* (%)	106 (18.8)	31 (18.5)	75 (18.9)	0.3255
Current smoker, *n* (%)	72 (12.5)	18 (10.8)	54 (13.3)	0.4134
Diabetes mellitus, *n* (%)	116 (19.9)	43 (25.0)	73 (17.6)	0.0321
3+ moderate/severe comorbidities *, *n* (%)	109 (18.6)	39 (22.9)	70 (16.9)	0.0867
Family history of prostate cancer, *n* (%)	76 (13.3)	22 (12.9)	20 (13.2)	0.9406
T staging at diagnosis, *n* (%)				0.1748
T1	194 (34.8)	55 (33.7)	139 (35.2)	
T2	266 (47.7)	86 (52.8)	180 (45.6)	
T3 or T4	98 (17.6)	22 (13.5)	76 (19.2)	
Gleason score at diagnosis, *n* (%)				0.781
≤6	203 (35.0)	53 (31.9)	150 (36.2)	
3 + 4	151 (26.0)	44 (26.5)	107 (25.9)	
4 + 3	93 (16.0)	29 (17.5)	64 (15.5)	
≥8	133 (22.9)	40 (24.1)	93 (22.5)	
PSA at diagnosis, ng/mL, median (Q1, Q3)	7.9 (5.4, 11.5)	7.8 (5.4, 11)	7.9 (5.4, 11.8)	0.8856
D’Amico risk class, *n* (%)				0.8596
Low	80 (13.9)	21 (12.7)	59 (14.4)	
Intermediate	235 (40.8)	69 (41.6)	166 (40.5)	
High	261 (45.3)	76 (45.8)	185 (45.1)	
RT method, *n* (%)				<0.0001
IGRT	423 (74.7)	101 (61.6)	322 (80.1)	
Non-IGRT	143 (25.3)	63 (38.4)	80 (19.9)	
RT technique, *n* (%)				0.1615
3D-CRT	186 (32.8)	61 (36.5)	125 (31.3)	
IMRT	229 (40.4)	56 (33.5)	173 (43.3)	
VMAT	143 (25.2)	48 (28.7)	95 (23.8)	
SBRT	9 (1.5)	2 (1.2)	7 (1.8)	
Volume treated with RT, *n* (%)				0.3743
Prostate alone	143 (25.9)	49 (29.9)	94 (24.2)	
Prostate plus seminal vesicles	324 (58.6)	91 (55.5)	233 (59.9)	
Prostate, seminal vesicles and pelvic nodes	86 (15.5)	24 (14.6)	62 (15.9)	
Dose Gy, *n* (%)				0.6125
<70	105 (21.3)	33 (22.0)	72 (21.0)	
70–75	210 (42.6)	59 (39.3)	151 (44.0)	
>75	178 (36.1)	58 (38.7)	120 (35.0)	
Association RT and ADT, *n* (%)				0.8388
Only RT	333 (57.9)	100 (58.8)	241 (58.4)	
ADT before or during RT	107 (18.6)	33 (19.4)	74 (17.9)	
ADT after RT	135 (23.5)	37 (21.8)	98 (23.7)	

SD: Standard Deviation; BMI: Body Mass Index; Q1: Quartile 1; Q3: Quartile 3; RT: Radiotherapy; IGRT: Image-Guided Radiation Therapy; 3D-CRT: three-dimensional conformal radiotherapy; IMRT: Intensity-Modulated Radiation Therapy; VMAT: Volumetric Modulated Arc Therapy; SBRT: Stereotactic body radiotherapy; * Based on Cumulative Illness Rating Scale (CIRS).

## Data Availability

The Pros-IT CNR participating data are available only to the collaborating scientists within the study.

## Supplementary Materials

The following are available online at https://www.mdpi.com/2072-6694/13/6/1479/s1, Table S1: Characteristics at diagnosis of patients according to trajectories identified for BF (sensitivity analysis, only patients completing the 24-month follow-up); Table S2: Univariate and multivariable logistic regression models to identify patients’ characteristics associated with BF trajectories; Table S3: Characteristics at diagnosis of patients according to trajectories identified for BB (sensitivity analysis, only patients completing the 24-month follow-up); Table S4: Univariate and multivariable* logistic regression models to identify patients’ characteristics associated with BB trajectories.

## References

[1] Buglione M., Noale M., Bruni A., Antonelli A., Bertoni F., Corvo’ R., Ricardi U., Borghetti P., Maddalo M., Simeone C. (2019). Treatment paths for localised prostate cancer in Italy: The results of a multidisciplinary, observational, prospective study (Pros-IT CNR). PLoS ONE.

[2] Vernooij R.W.M., Cremers R.G.H.M., Jansen H., Somford D.M., Kiemeney L.A., van Andel G., Wijsman B.P., Busstra M.B., van Moorselaar R.J.A., Wijnen E.M. (2020). Urinary incontinence and erectile dysfunction in patients with localized or locally advanced prostate cancer: A nationwide observational study. Urol. Oncol..

[3] Hoffman K.E., Penson D.F., Zhao Z., Huang L.C., Conwill R., Laviana A.A., Joyce D.D., Luckenbaugh A.N., Goodman M., Hamilton A.S. (2020). Patient-Reported Outcomes Through 5 Years for Active Surveillance, Surgery, Brachytherapy, or External Beam Radiation With or Without Androgen Deprivation Therapy for Localized Prostate Cancer. JAMA.

[4] Budaus L., Bolla M., Bossi A., Cozzarini C., Crook J., Widmark A., Wiegel T. (2012). Functional outcomes and complications following radiation therapy for prostate cancer: A critical analysis of the literature. Eur. Urol..

[5] Barocas D.A., Alvarez J., Resnick M.J., Koyama T., Hoffman K.E., Tyson M.D., Conwill R., McCollum D., Cooperberg M.R., Goodman M. (2017). Association Between Radiation Therapy, Surgery, or Observation for Localized Prostate Cancer and Patient-Reported Outcomes After 3 Years. JAMA.

[6] Ram N., Grimm K.J. (2009). Growth Mixture Modeling: A method for identifying differences in longitudinal change among unobserved groups. Int. J. Behav. Dev..

[7] Palumbo C., Bruni A., Antonelli A., Artibani W., Bassi P.F., Bertoni F., Borghetti P., Bracarda S., Cicchetti A., Corvò R. (2021). Health-related quality of life 24-month after prostate cancer diagnosis: An update from the Pros-IT CNR prospective observational study. Health-related quality of life 24-month after prostate cancer diagnosis: An update from the Pros-IT CNR prospective observational study. Minerva Urol. Nefrol..

[8] Noale M., Maggi S., Artibani W., Bassi P.F., Bertoni F., Bracarda S., Conti G.N., Corvò R., Gacci M., the Pros-IT CNR study group (2017). Pros-IT CNR: An Italian prostate cancer monitoring project. Aging Clin. Exp. Res..

[9] Porreca A., Noale M., Artibani W., Bassi P.F., Bertoni F., Bracarda S., Conti G.N., Corvò R., Gacci M., the Pros-IT CNR study group (2018). Disease-specific and general health-related quality of life in newly diagnosed prostate cancer patients: The Pros-IT CNR study. Health Qual. Life Outcomes.

[10] Gacci M., Noale M., Artibani W., Bassi P.F., Bertoni F., Bracarda S., Conti G.N., Corvò R., Graziotti P., Magrini S.M. (2017). Quality of Life After Prostate Cancer Diagnosis: Data from the Pros-IT CNR. Eur. Urol. Focus.

[11] Gacci M., Livi L., Paiar F., Detti B., Litwin M., Bartoletti R., Giubilei G., Cai T., Mariani M., Carini M. (2005). Quality of life after radical treatment of prostate cancer: Validation of the Italian version of the University of California-Los Angeles Prostate Cancer Index. Urology.

[12] Conwell Y., Forbes N.T., Cox C., Caine E.D. (1993). Validation of a measure of physical illness burden at autopsy: The Cumulative Illness Rating Scale. J. Am. Geriatr. Soc..

[13] Jones B.L., Nagin D.S., Roeder K. (2001). A SAS procedure based on mixture models for estimating developmental trajectories. Sociol. Methods Res..

[14] Andruff H., Carraro N., Thompson A., Gaudreau P., Louvet P. (2009). Latent class growth modelling: A tutorial. Tutor. Quant. Methods Psychol..

[15] Geinitz H., Thamm R., Scholz C., Heinrich C., Prause N., Kerndl S., Keller M., Busch R., Molls M., Zimmermann F.B. (2010). Longitudinal analysis of quality of life in patients receiving conformal radiation therapy for prostate cancer. Strahlenther Onkol..

[16] Antonelli A., Palumbo C., Noale M., Artibani W., Bassi P., Bertoni F., Bracarda S., Bruni A., Corvò R., Gacci M. (2020). Overview of potential determinants of radical prostatectomy versus radiation therapy in management of clinically localized prostate cancer: Results from an Italian, prospective, observational study (the Pros-IT CNR study). Minerva Urol. Nefrol..

[17] Hamstra D.A., Conlon A.S., Daignault S., Dunn R.L., Sandler H.M., Hembroff A.L., Zietman A.L., Kaplan I., Ciezki J., Kuban D.A. (2013). Multi-institutional prospective evaluation of bowel quality of life after prostate external beam radiation therapy identifies patient and treatment factors associated with patient-reported outcomes: The PROSTQA experience. Int J. Radiat. Oncol. Biol. Phys..

[18] Parinaz M., Behzad B., Fatemeh V., Sousan N. (2020). Functional response difference between diabetic/normal cancerous patients to inflammatory cytokines and oxidative stresses after radiotherapy. Rep. Pract. Oncol. Radiother.

[19] Özkan E.E., Erdemoğlu E., Raoufi J. (2019). Turk Impact of diabetes on gastrointestinal and urinary toxicity after radiotherapy for gynecologic malignancy. J. Obstet. Gynecol..

[20] Alashkham A., Paterson C., Hubbard S., Nabi G. (2017). What is the impact of diabetes mellitus on radiation induced acute proctitis after radical radiotherapy for adenocarcinoma prostate? A prospective longitudinal study. Clin. Transl. Radiat. Oncol..

[21] Murray J., Griffin C., Gulliford S., Syndikus I., Staffurth J., Panades M., Scrase C., Parker C., Khoo V., Dean J. (2020). CHHiP Investigators A randomised assessment of image guided radiotherapy within a phase 3 trial of conventional or hypofractionated high dose intensity modulated radiotherapy for prostate cancer. Radiother Oncol..

[22] Mangar S.A., Huddart R.A., Parker C.C., Dearnaley D.P., Khoo V.S., Horwich A. (2005). Technological advances in radiotherapy for the treatment of localised prostate cancer. Eur. J. Cancer.

[23] Chung H.T., Xia P., Chan L.W., Park-Somers E., Roach M. (2009). Does imageguided radiotherapy improve toxicity profile in whole pelvic-treated high-risk prostate cancer? Comparison between IG-IMRT and IMRT. Int. J. Radiat. Oncol. Biol. Phys..

[24] De Crevoisier R., Bayar M.A., Pommier P., Muracciole X., Pêne F., Dudouet P., Latorzeff I., Beckendorf V., Bachaud J.-M., Laplanche A. (2018). Daily versus weekly prostate cancer image guided radiation therapy: Phase 3 multicenter randomised trial. Int. J. Radiat. Oncol. Biol. Phys..

[25] Tøndel H., Lund J.-Å., Lydersen S., Wanderås A.D., Aksnessæther B., Jensen C.A., Kaasa S., Solberg A. (2018). Radiotherapy for prostate cancer—Does daily image guidance with tighter margins improve patient reported outcomes compared to weekly orthogonal verified irradiation? Results from a randomized controlled trial. Radiother Oncol..

