# Supplementary material for: Impact of Gastrointestinal Side Effects on Patients’ Reported Quality of Life Trajectories after Radiotherapy for Prostate Cancer: Data from the Prospective, Observational Pros-IT CNR Study
Source: Cancers (Basel). 2021 Mar 23;13(6):1479. doi: 10.3390/cancers13061479 (PMC8004900; doi:10.3390/cancers13061479)
Supplement: Supplementary file 1 [file cancers-13-01479-s001.pdf]

Table S1. Characteristics at diagnosis of patients according to trajectories identified for BF (sensitivity analysis, only patients completing the 24-month follow-up)

	**Trajectory 1 (n=40)**	**Trajectory 2 (n=103)**	**Trajectory 3 (n=264)**	**p-value**
Age at diagnosis, years, mean ± SD	71.7±5.4	72.1±6.3	72.8±5.1	0.4927
Education > lower secondary school, n (%)	14 (35.0)	46 (45.1)	113 (43.8)	0.5266
BMI ≥ 30 kg/m^2, n (%)	10 (25.0)	21 (20.8)	42 (16.6)	0.6985
Current smoker, n (%)	3 (7.5)	14 (13.9)	33 (12.8)	0.5762
Diabetes mellitus, n (%)	11 (27.5)	20 (19.4)	47 (17.8)	0.3538
3+ moderate/severe comorbidities*, n (%)	16 (40.0)	20 (19.6)	37 (14.0)	0.0003
Family history of prostate cancer, n (%)	4 (10.0)	15 (14.7)	33 (12.6)	0.7342
T staging at diagnosis, n (%)				0.3130
T1	11 (29.7)	36 (39.1)	91 (35.6)	
T2	22 (59.5)	44 (47.8)	115 (44.9)	
T3 or T4	4 (10.8)	12 (13.0)	50 (19.5)	
Gleason score at diagnosis, n (%)				0.8901
≤6	13 (33.3)	38 (36.9)	90 (34.6)	
3+4	9 (23.1)	24 (23.3)	73 (28.1)	
4+3	9 (23.1)	17 (16.5)	40 (15.4)	
≥8	8 (20.5)	24 (23.3)	57 (21.9)	
PSA at diagnosis, ng/mL, median (Q1, Q3)	8.6 (5.5, 11.6)	7.8 (5.2, 13.1)	7.9 (5.7, 11.0)	0.7721
D'Amico risk class, n (%)				0.8511
Low	4 (10.3)	17 (17.0)	39 (15.1)	
Intermediate	15 (38.5)	40 (40.0)	99 (38.2)	
High	20 (51.3)	43 (43.0)	121 (46.7)	
RT method, n (%)				0.0442
IGRT	25 (62.5)	71 (69.7)	194 (76.1)	
Non-IGRT	15 (37.5)	31 (30.3)	61 (23.9)	
RT technique, n (%)				0.0053
3D-CRT	20 (50.0)	39 (38.2)	75 (29.4)	
IMRT	9 (22.5)	34 (33.3)	112 (43.9)	
VMAT	11 (27.5)	24 (23.5)	66 (25.9)	
SBRT	0 (0.0)	5 (4.9)	2 (0.8)	
Volume treated with RT, n (%)				0.4201
Prostate alone	9 (22.5)	24 (23.8)	68 (26.9)	
Prostate plus seminal vesicles	22 (55.0)	65 (64.4)	140 (55.3)	
Prostate, seminal vesicles and pelvic nodes	9 (22.5)	12 (11.9)	45 (17.8)	
Dose Gy, n (%)				0.4076
<70	4 (10.5)	21 (23.1)	50 (23.4)	
70-75	18 (47.4)	26 (39.6)	94 (43.9)	
>75	16 (42.1)	34 (37.4)	70 (32.7)	
Association RT and ADT, n (%)				0.4336
Only RT	28 (70.0)	56 (54.4)	158 (60.3)	
ADT before or during RT	6 (15.0)	17 (16.5)	43 (16.4)	
ADT after RT	6 (15.0)	30 (29.1)	61 (23.3)	

Table S2. Univariate and multivariable* logistic regression models to identify patients' characteristics associated with BF trajectories

	Univariate models						Multivariable model*					
	Trajectory 2 vs 3			Trajectory 1 vs 3			Trajectory 2 vs 3			Trajectory 1 vs 3		
	**OR**	**95% CI**	**p-value**	**OR**	**95% CI**	**p-value**	**OR**	**95% CI**	**p-value**	**OR**	**95% CI**	**p-value**
Age at diagnosis, years	0.99	0.95-1.02	0.3916	0.97	0.92-1.01	0.1714						
Education > lower secondary school vs higher	1.01	0.68-1.50	0.9756	1.10	0.63-1.91	0.7420						
BMI ≥ 30 kg/m^2	1.32	0.80-2.16	0.2778	1.47	0.77-2.84	0.2460						
Current smoker vs	1.14	0.64-2.05	0.6553	0.97	0.41-2.25	0.9346						
Diabetes mellitus	1.16	0.71-1.89	0.5561	1.45	0.77-2.74	0.2551						
3+ moderate/severe comorbidities* vs ≤2	1.61	0.98-2.64	0.0619	3.33	1.84-6.04	<0.0001	1.67	0.99-2.83	0.0560	3.80	2.04-7.08	<0.0001
Family history of prostate cancer	1.22	0.70-2.14	0.4864	0.91	0.39-2.12	0.8286						
D'Amico risk class												
High vs Low	0.91	0.50-1.64	0.7413	1.33	0.52-3.40	0.7157						
Intermediate vs Low	0.89	0.49-1.63	0.5527	1.49	0.58-3.81	0.4060						
RT method Non-IGRT vs IGRT	1.35	0.86-2.11	0.1980	2.00	1.12-3.57	0.0190						
RT technique, 3D-CRT vs IMRT, VMAT	1.42	0.93-2.18	0.1039	2.07	1.19-3.59	0.0102	1.47	0.94-2.28	0.0881	2.17	1.23-3.87	0.0088
Volume treated with RT												
Prostate plus seminal vesicles vs prostate alone	1.33	0.82-2.15	0.2461	1.20	0.62-2.34	0.5883						
Prostate, seminal vesicles, pelvic nodes vs prostate alone	0.83	0.41-1.66	0.5896	1.30	0.55-3.04	0.5505						
Dose Gy												
70-75 vs <70	0.80	0.46-1.40	0.4349	1.87	0.77-4.52	0.1641						
>75 vs <70	1.20	0.69-2.10	0.5190	2.42	0.99-5.88	0.0523						
Association RT and ADT												
ADT before/during RT vs only RT	1.02	0.60-1.74	0.9351	0.76	0.36-1.59	0.4639						
ADT after RT vs only RT	1.29	0.81-2.06	0.2795	0.56	0.26-1.20	0.1357						

OR: Odds Ratio; CI: Confidence Interval

*stepwise selection procedure with p-value to entry=0.10 and p-value to stay=0.20. Variables considered as possible independent variables: age at diagnosis, education, marital status, family history of prostate cancer, comorbidities, diabetes, body mass index (BMI), prostate-specific antigen (PSA) at diagnosis, Gleason score, clinical T-Stage and characteristics of RT (method, Image-Guided Radiation Therapy (IGRT) vs non-IGRT; technique, three-dimensional conformal radiotherapy (3D-CRT) vs Intensity-Modulated Radiation Therapy (IMRT), Volumetric Modulated Arc Therapy (VMAT) and Stereotactic body radiotherapy (SBRT); the volume treated, prostate alone vs prostate plus seminal vesicles vs prostate, seminal vesicles and pelvic nodes) and association with ADT treatments (only RT, ADT before RT, ADT after RT). ** Based on Cumulative Illness Rating Scale (CIRS)

Table S3. Characteristics at diagnosis of patients according to trajectories identified for BB (sensitivity analysis, only patients completing the 24-month follow-up)

	Trajectory 1 (n=93)	Trajectory 2 (n=308)	p-value
Age at diagnosis, years, mean ± SD	72.4±6.2	72.6±5.2	0.9055
Education > lower secondary school, n (%)	44 (47.3)	126 (41.7)	0.3411
BMI ≥ 30 kg/m^2, n (%)	19 (20.4)	54 (18.2)	0.7916
Current smoker, n (%)	8 (8.9)	42 (13.9)	0.2139
Diabetes mellitus, n (%)	25 (26.9)	53 (17.3)	0.0403
3+ moderate/severe comorbidities*, n (%)	25 (26.9)	48 (15.6)	0.0139
Family history of prostate cancer, n (%)	12 (12.9)	39 (12.8)	0.9766
T staging at diagnosis, n (%)			0.0143
T1	26 (29.6)	109 (37.5)	
T2	53 (60.2)	126 (43.3)	
T3 or T4	9 (10.2)	56 (19.2)	
Gleason score at diagnosis, n (%)			0.5411
≤6	26 (28.6)	111 (36.4)	
3+4	26 (28.6)	79 (25.9)	
4+3	18 (19.8)	48 (15.7)	
≥8	21 (23.1)	67 (22.0)	
PSA at diagnosis, ng/mL, median (Q1, Q3)	7.8 (5.7, 10.3)	8.1 (5.4, 12.0)	0.5239
D’Amico risk class, n (%)			0.2729
Low	9 (10.0)	50 (16.6)	
Intermediate	35 (39.9)	117 (38.7)	
High	46 (51.1)	135 (44.7)	
RT method, n (%)			0.0005
IGRT	52 (58.4)	233 (77.2)	
Non-IGRT	37 (41.6)	69 (22.9)	
RT technique, n (%)			0.3107
3D-CRT	35 (38.5)	97 (32.3)	
IMRT	29 (31.9)	126 (42.0)	
VMAT	26 (28.6)	71 (23.7)	
SBRT	1 (1.1)	6 (2.0)	
Volume treated with RT, n (%)			0.9632
Prostate alone	23 (25.6)	75 (25.2)	
Prostate plus seminal vesicles	53 (58.9)	173 (58.1)	
Prostate, seminal vesicles and pelvic nodes	14 (15.6)	50 (16.8)	
Dose Gy, n (%)			0.7291
<70	19 (22.6)	55 (21.7)	
70-75	33 (39.3)	112 (44.1)	
>75	32 (38.1)	87 (34.2)	
Association RT and ADT, n (%)			0.5161
Only RT	51 (54.8)	187 (61.1)	
ADT before or during RT	18 (19.4)	47 (15.4)	
ADT after RT	24 (25.8)	72 (23.5)	

Table S4. Univariate and multivariable* logistic regression models to identify patients' characteristics associated with BB trajectories

	Univariate models Trajectory 1 vs 2			Multivariable model* Trajectory 1 vs 2		
	OR	95% CI	p-value	OR	95% CI	p-value
Age at diagnosis, years	0.98	0.95-1.01	0.2789			
Education > lower secondary school vs higher	0.75	0.52-1.08	0.1162			
BMI ≥ 30 kg/m^2	0.97	0.61-1.55	0.9031			
Current smoker	0.79	0.45-1.39	0.4143			
Diabetes mellitus	1.59	1.04-2.45	0.0329	1.69	1.06-2.67	0.0269
3+ moderate/severe comorbidities* vs ≤2	1.47	0.95-2.28	0.0878			
Family history of prostate cancer	0.98	0.58-1.67	0.9407			
D'Amico risk class						
High vs Low	1.15	0.66-2.03	0.5948			
Intermediate vs Low	1.17	0.66-2.07	0.6190			
RT method non-IGRT vs IGRT	2.51	1.69-3.74	<0.0001	2.57	1.70-3.86	<0.0001
RT technique, 3D-CRT vs IMRT, VMAT	1.26	0.86-1.84	0.2382			
Volume treated with RT						
Prostate plus seminal vesicles vs prostate alone	1.08	0.67-1.72	0.7647			
Prostate, seminal vesicles, pelvic nodes vs prostate alone	0.91	0.58-1.42	0.6770			
Dose Gy						
70-75 vs <70	0.85	0.51-1.42	0.5399			
>75 vs <70	1.06	0.63-1.77	0.8406			
Association RT and ADT						
ADT before/during RT vs only RT	0.75	0.49-1.14	0.1798			
ADT after RT vs only RT	0.74	0.41-1.33	0.3180			

OR: Odds Ratio; CI: Confidence Interval

*stepwise selection procedure with p-value to entry=0.10 and p-value to stay=0.20. Variables considered as possible independent variables: age at diagnosis, education, marital status, family history of prostate cancer, comorbidities, diabetes, body mass index (BMI), prostate-specific antigen (PSA) at diagnosis, Gleason score, clinical T-Stage and characteristics of RT (method, Image-Guided Radiation Therapy (IGRT) vs non-IGRT; technique, three-dimensional conformal radiotherapy (3D-CRT) vs Intensity-Modulated Radiation Therapy (IMRT), Volumetric Modulated Arc Therapy (VMAT) and Stereotactic body radiotherapy (SBRT); the volume treated, prostate alone vs prostate plus seminal vesicles vs prostate, seminal vesicles and pelvic nodes) and association with ADT treatments (only RT, ADT before RT, ADT after RT)

** Based on Cumulative Illness Rating Scale (CIRS)
